# The value of four stage vestibular hydrops grading and asymmetric perilymphatic enhancement in the diagnosis of Menière’s disease on MRI

**DOI:** 10.1007/s00234-019-02155-7

**Published:** 2019-02-05

**Authors:** Anja Bernaerts, Robby Vanspauwen, Cathérine Blaivie, Joost van Dinther, Andrzej Zarowski, Floris L. Wuyts, Stephanie Vanden Bossche, Erwin Offeciers, Jan W. Casselman, Bert De Foer

**Affiliations:** 1 Department of Radiology, GZA Hospitals Antwerp, Oosterveldlaan 24, 2610 Wilrijk, Belgium; 2 European institute for ORL-HNS, GZA Hospitals Antwerp, Oosterveldlaan 24, 2610 Wilrijk, Belgium; 3 Antwerp University research center for Equilibrium and Aerospace, Wilrijkstraat 10, Building N012, 2610 Wilrijk, Belgium; 4 Department of Radiology, AZ Jan Palfijn Ghent AV, Watersportlaan 5, 9000 Ghent, Belgium; 5 Department of Radiology, AZ Sint-Jan Hospital AV, Ruddershove 10, 8000 Bruges, Belgium

**Keywords:** Magnetic resonance imaging, Menière’s disease, Endolymphatic hydrops, Perilymph, Classification, Diagnosis

## Abstract

**Purpose:**

There is still a clinical-radiologic discrepancy in patients with Menière’s disease (MD). Therefore, the purpose of this study was to investigate the reliability of current MRI endolymphatic hydrops (EH) criteria according to Baráth in a larger study population and the clinical utility of new imaging signs such as a supplementary fourth low-grade vestibular EH and the degree of perilymphatic enhancement (PE) in patients with Menière’s disease (MD).

**Methods:**

This retrospective study included 148 patients with probable or definite MD according to the 2015 American Academy of Otolaryngology, Head and Neck Surgery criteria who underwent a 4-h delayed intravenous Gd-enhanced 3D-FLAIR MRI between January 2015 and December 2016. Vestibular EH, vestibular PE, cochlear EH, and cochlear PE were reviewed twice by three experienced readers. Cohen’s Kappa and multivariate logistic regression were used for analysis.

**Results:**

The intra- and inter-reader reliability for the grading of vestibular-cochlear EH and PE was excellent (0.7 < kappa < 0.9). The two most distinctive characteristics to identify MD are cochlear PE and vestibular EH which combined gave a sensitivity and specificity of 79.5 and 93.6%. By addition of a lower grade vestibular EH, the sensitivity improved to 84.6% without losing specificity (92.3%). Cochlear EH nor vestibular PE showed added-value.

**Conclusions:**

MRI using vestibular-cochlear EH and PE grading system is a reliable technique. A four-stage vestibular EH grading system in combination with cochlear PE assessment gives the best diagnostic accuracy to detect MD.

## Introduction

More than 150 years ago, Prosper Menière described for the first time that vertigo can be elicited from the inner ear. Until then, it was believed vertigo was a disorder of the brain. The pathology named after him is characterized by a clinical triad of fluctuating low-frequency sensorineural hearing loss, tinnitus and/or aural fullness, and vertigo attacks lasting for at least 20 min. In 1995, the American Academy of Otolaryngology - Head and Neck Surgery (AAO HNS) established a specific set of criteria for the diagnosis of Menière’s disease (MD). In this classification, the disease is divided into certain (with postmortem histologic confirmation), definite, probable, and possible categories [1]. In 2015, the Bárány society formulated simplified diagnostic criteria for MD, including only two categories: definite MD and probable MD [2]. Unfortunately, in clinical practice, patients rarely present as textbook cases. Especially in the first years, the symptoms may be transient and incomplete. In about 9% of cases, the time period between the onset of vertigo and hearing loss can last more than 10 years [3]. In the advanced stage of Menière’s disease (MD), the symptoms are permanent. The clinical diagnosis of MD can be complemented with a battery of audiological, vestibular, and electrophysiological tests; however, the lack of a definitive gold standard diagnostic test still complicates the process of diagnosis [3].

Until recently, the role of imaging has been to exclude other pathologies mimicking MD, such as schwannomas, labyrinthitis, intracranial liquor hypotension, and so on. However, at present, the development of delayed gadolinium (Gd)-enhanced high-resolution magnetic resonance imaging (MRI) of the inner ear has enabled us to visualize the histopathological basis of MD, namely the endolymphatic hydrops (EH) as described by Hallpike in 1938 on temporal bone histologic studies [4]. Four-hours delayed intravenous contrast-enhanced three-dimensional fluid attenuated inversion recovery (3D FLAIR) MRI has become a validated technique for demonstration of this EH by allowing dilute contrast to accumulate within the perilymphatic compartment, where the blood-labyrinth barrier is permeable, outlining the impermeable endolymphatic compartment [5].

Nevertheless, it is reported that 10–33% of patients with MD do not have MRI-demonstrable changes of hydrops [6–8]. This clinical-radiologic discrepancy still reflects an incomplete understanding of the disease process and the need for additional imaging biomarkers of disease activity in MD beyond EH. The purpose of this study is to investigate the reliability and validity of the current MRI diagnostic criteria and grading system for EH [8] on delayed intravenous contrast-enhanced 3D FLAIR MRI in a larger study population and the clinical utility of possible new imaging signs such as a supplementary fourth low-grade vestibular EH and the degree of perilymphatic enhancement (PE).

## Materials and methods

### Patients

Between January 2015 and December 2016, 256 consecutive patients with Menièriform symptoms such as tinnitus, vertigo, aural fullness, fluctuating hearing loss, or a combination of these symptoms were referred for 3 T MRI of the temporal bone to demonstrate EH. All patients with a history of previous ear surgery and other vestibular or central disorders were excluded from the study. With institutional approval for the study (GZA study number: 161205RETRO), the MRI data of the remaining 148 patients (296 ears) were retrospectively analyzed.

Patients were clinically evaluated by our ORL-HNS department and were diagnosed with probable or definite MD according to the 2015 revised diagnostic criteria of the AAO HNS. The classification of the clinical symptoms and functional tests was done independently by a dedicated otorhinolaryngologist and vestibular clinical scientist (RV and CB with and experience of, respectively, 7 and 11 years).

### MRI protocol

All MRI were performed on a 3-T scanner (MAGNETOM Skyra-Fit, Siemens, Erlangen, Germany) using a 32-channel array head coil. MRI was performed 4 h after a double dose of intravenous Gd administration to assure the maximum perilymphatic enhancement, according to previous reports in literature [5] (Gadovist; Bayer-Schering Pharma, Berlin, Germany; 1.0 mmol/mL at a dose of 0.2 mmol/kg). A 3D FLAIR sequence was performed with the following parameters: FOV, 190 mm; section thickness, 0.8 mm; TR, 6000 ms; TE, 168 ms; number of excitations, 1; TI, 2000 ms; flip angle, 180°; matrix, 384 # 384; bandwidth, 186 Hz/pixel; turbo factor, 27; voxel size, 0.5 × 0.5 × 0.8; and scan time, 14 min. No patients had to be excluded from the study due to insufficient image quality.

The MR images were qualitatively analyzed twice by three experienced head and neck radiologists (AB, BDF and JC, with an experience in head and neck radiology of, respectively, 15, 25, and 35 years) blinded to the clinical findings and the side, uni- or bilaterality of symptoms. The time interval between the two readings was 2–4 weeks.

### Imaging analysis

**Fig. 1:**
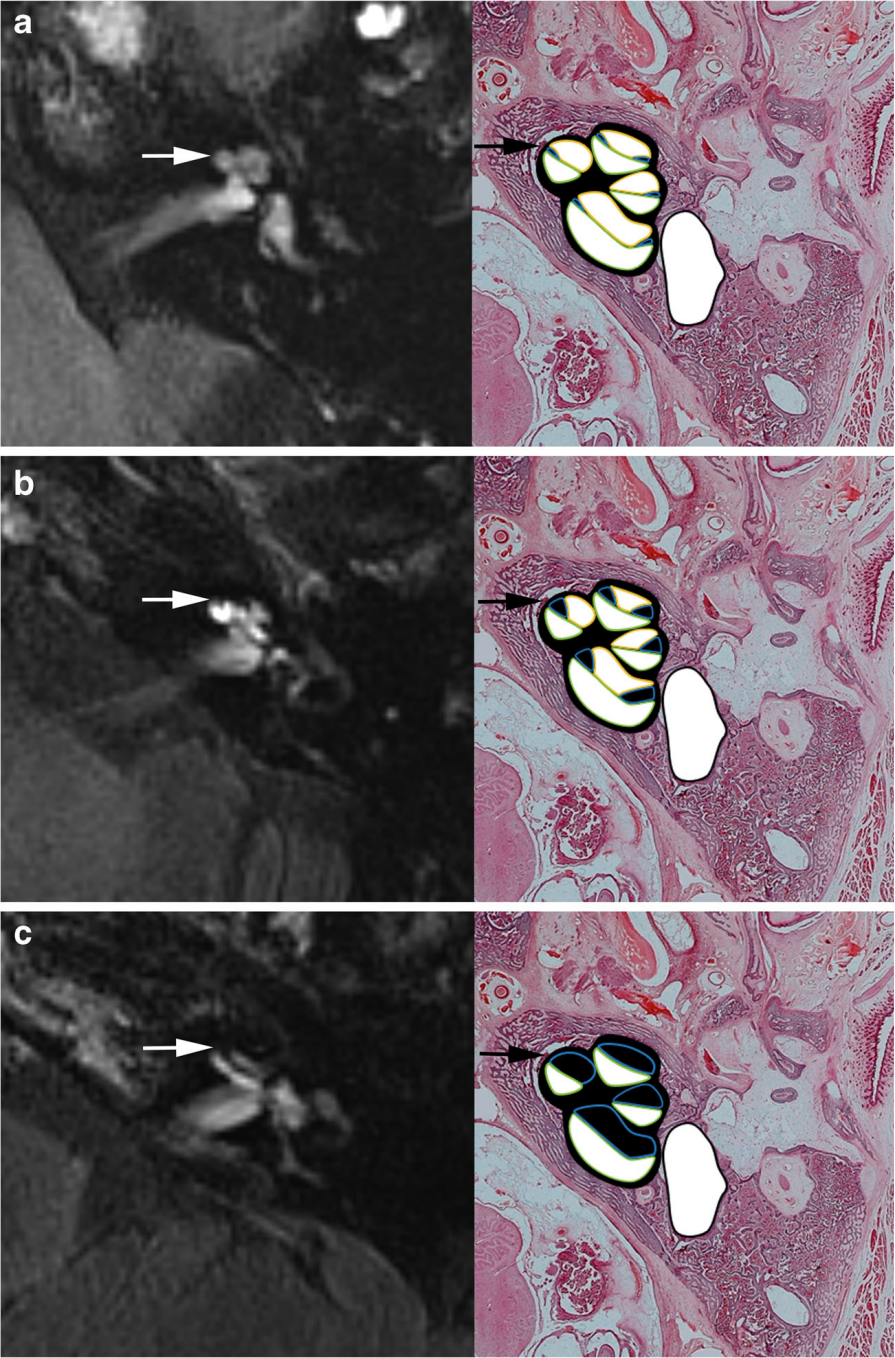
Cropped axial delayed gadolinium-enhanced 3D FLAIR images at midmodiolar area of the cochlea and correlating axial cryosections with hematoxylin and eosin staining (magnification, × 7) and color overlay. **a** Normal cochlea: In the normal cochlea, one can recognize the interscalar septum (arrow), the scala tympani, and scala vestibuli. The scala media is normally minimally visible. **b** Cochlear hydrops grade I: The scala media becomes indirect visible as a nodular black cut-out of the scala vestibuli (arrow). **c** Cochlear hydrops grade II: The scala vestibuli (arrow) is fully obliterated due to the distended cochlear duct

The degree of EH in the vestibule and cochlea was assessed by visual comparison of the relative areas of the non-enhanced endolymphatic space versus the contrast-enhanced perilymph space in the axial plane, separately for the cochlea and the vestibule. The degree of cochlear hydrops was categorized as none, grade I, or grade II according to the criteria previously described by Baráth et al. [8] (Fig. 1).

**Fig. 2:**
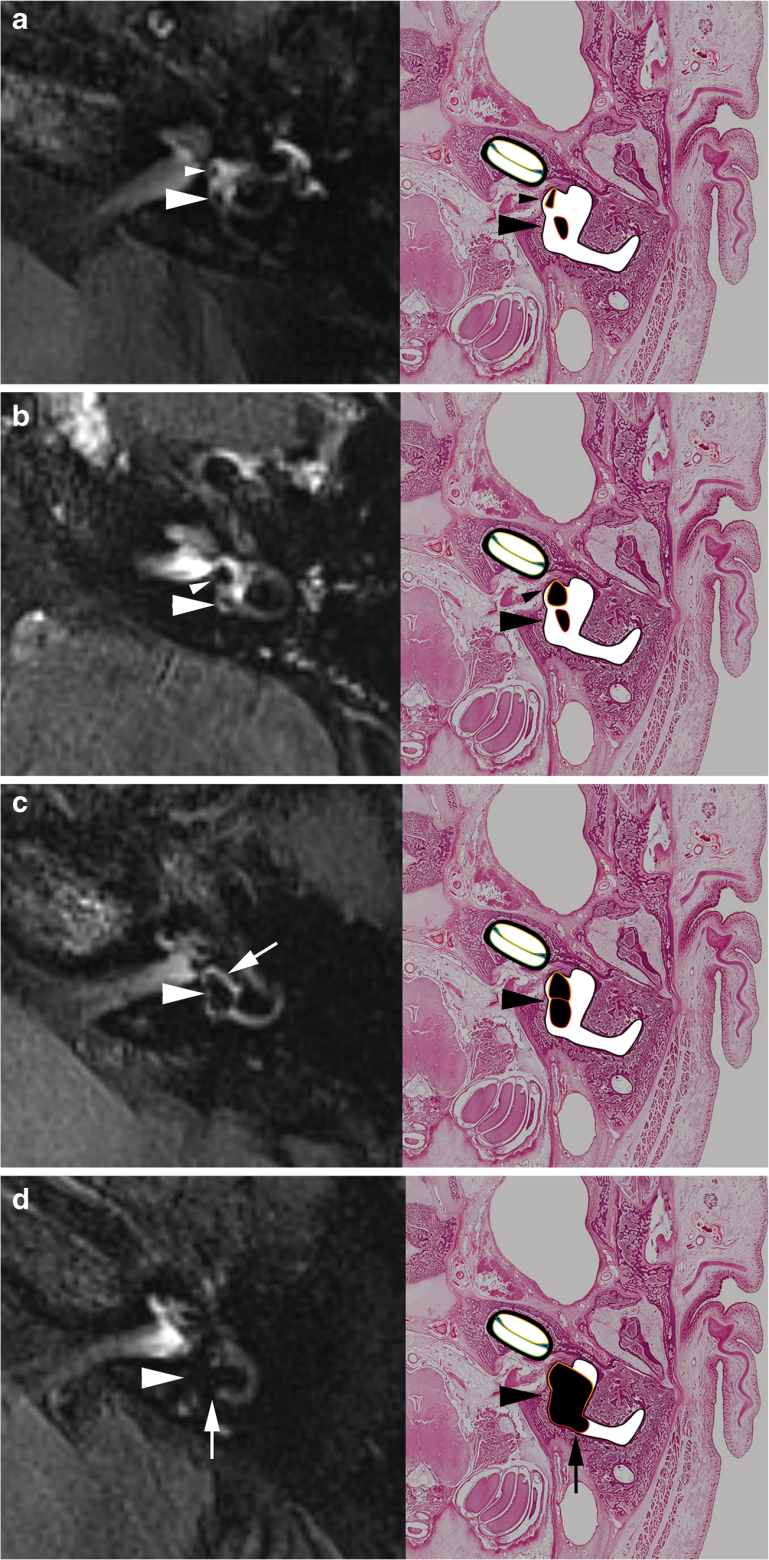
Cropped axial delayed gadolinium-enhanced 3D FLAIR images at the inferior part of the vestibulum and correlating axial cryosections with hematoxylin and eosin staining (magnification, × 7) and color overlay. **a** Normal vestibule: The saccule (small arrowhead) and utricle (large arrowhead) are visibly separately and take less than half of the surface of the vestibule. **b** Vestibular hydrops grade I: The saccule (small arrowhead), normally the smallest of the two vestibular sacs, has become equal or larger than the utricle (large arrowhead) but is not yet confluent with the utricle. **c** Vestibular hydrops grade II: There is a confluence of the saccule and utricle (arrowhead) with still a peripheral rim enhancement of the perilymphatic space (arrow). **d** Vestibular hydrops grade III: The perilymphatic enhancement is no longer visible (arrowhead). There is a full obliteration of the bony vestibule. Also notice in this case, the beginning utricular protrusion in the non-ampullated part of the LSCC (arrow)

However, for the degree of the vestibular hydrops, we used a modified grading system, as in our experience there were patients with subtle abnormalities who were categorized as normal according to the three-stage grading system of Baráth. We added a lower grade I vestibular hydrops in which the saccule, normally the smallest of the two vestibular sacs, became equal or larger than the utricle but is not yet confluent with the utricle. In this modified four-stage grading system, the Baráth grade I became grade II, and the Baráth grade II became grade III (Fig. 2). The visual assessment of the saccule-to-utricle ratio was done on the lowest axial images at the inferior part of the vestibule as, according to histological studies, the saccule occupies the inferior, medial, and anterior part of the vestibule [9].

**Fig. 3:**
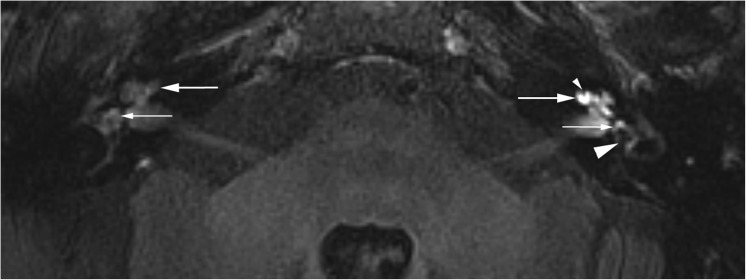
Axial delayed gadolinium-enhanced 3D FLAIR images at the level of the inner ear in a 77-year-old woman with unilateral left-sided definite MD and cochlear hydrops grade I (small arrowhead) and vestibular hydrops grade II according to the four-stage grading system (large arrowhead). Note increased vestibular (small arrow) and cochlear (large arrow) perilymphatic enhancement (PE) on the symptomatic side compared with the normal right labyrinth. This is the signature of BPB-impairment

The degree of PE was also evaluated semi-quantitatively in all ears by visually comparing the degree of enhancement of the concerning ear with the contralateral ear. The degrees of enhancement both for the vestibule and cochlea were classified separately into three groups: less, equal, or more (Fig. 3). In case of a grade 3 vestibular hydrops, the evaluation of the vestibular PE is considered as non-applicable since there is no visible perilymphatic space left to evaluate.

### Statistical analysis

Vestibular EH, vestibular PE, cochlear EH, and cochlear PE were reviewed twice by three readers (AB, BDF and JC). The Cohen's kappa statistic was used to estimate the degree of chance-adjusted agreement between the readers. In addition, for each parameter, the mode, i.e., the most common chosen value among the six different readings (three readers which each read two times), was taken as the “truth” and was linked to the clinical findings. In case of an ex aequo between the six readings, the images were discussed with the three radiologists to reach a consensus. Next, stepwise logistic regression analysis was done to identify the essential radiological predicting factors leading to the most optimal sensitivity and specificity for the correct identification of MD ears versus control ears. Both forward and backward regressions were used. Duration of MD was correlated with the different MRI variables by means of Spearman correlation analysis.

Further results were reported by means of cross-tabulations and comparing descriptive statistics like mean, standard deviation, median, first and third quartiles, minimum and maximum between the different classifications. IBM SPSS Statistics V24 was used for the statistical analysis.

## Results

Of the cohort of 148 patients, 105 patients had unilateral disease resulting in 105 normal ears, 26 undefined (not enough data) ears, 1 probable MD ear, and 78 definite MD ears. Thirteen patients had bilateral disease resulting in 5 undefined (not enough data) ears, 5 probable MD ears, and 16 definite MD ears. Thirty patients were classified with undefined problems. The average age (standard deviation) of the cohort was 56 (12) years (range, 25–84 years), with a female-to-male ratio of 81:67.

For the assessment of the intra- and inter-reader reliability of the readings by the radiologists by means of Cohen’s kappa analysis, all 148 cases were used. The intra-reader agreement was assessed by comparing per ear the first reading with the second reading for each individual reader in a test-retest design. This yields per parameter six kappa values. Table 1 reports the averaged kappa. In general, the kappa is excellent for all the measures (0.87 < kappa < 0.92). The inter-reader agreement was based on the agreement per ear for all three readers, calculated for the first reading and averaged. The agreement between the three readers was also high for every measurement (kappa 0.73–0.83) (Table 2). In parallel, the inter-reader agreement of the clinicians for assessing the diagnosis of MD (no, probable, definite) based on the clinical data yields a kappa (± se) = 0.66 ± 0.05.

**Table 1:** Mean kappa representing the intra-reader agreement based on 6 intra-reader kappa’s for the first and second assessment of the radiological data (3 authors and 2 ears)

	Vestibular EH	Cochlear EH	Vestibular PE	Cochlear PE
Intra-reader Mean Kappa	0.92	0.88	0.85	0.87
SE	0.02	0.03	0.04	0.04
Min	0.86	0.77	0.74	0.78
Max	0.97	0.96	0.93	0.98

*EH* endolymphatic hydrops, *PE* perilymphatic enhancement

**Table 2:** Mean kappa representing the inter-reader agreement based on all readings of the radiological data

	Vestibular EH	Cochlear EH	Vestibular PE	Cochlear PE
Inter-reader Mean Kappa	0.81	0.83	0.73	0.77
SE	0.03	0.03	0.03	0.04
Min	0.79	0.82	0.73	0.76
Max	0.83	0.83	0.73	0.77

*EH* endolymphatic hydrops, *PE* perilymphatic enhancement

For the logistic regression, to assess the ability of the imaging characteristics to predict MD, we used only patients with unilateral definite MD (78 patients). Their contralateral ears were normal and served as controls in this analysis. Patients with bilateral MD were not included, since the notion that the concerned diseased ear has more or less enhancement than the control ear becomes irrelevant. We obtained thereby 78 definite MD ears and 78 control ears. 17 (21%) of these definite MD ears received previous intratympanic therapy (ITT). Analysis was done both for the entire definite MD ears (78) and for the definite MD ears (61) without ITT.

Logistic regression was performed both for the three-stage hydrops grading system according to Baráth and the new four-stage hydrops grading system. To go from the four-stage to the three-stage grading system, grade 0 and grade I are combined to grade 0, grade II becomes grade I, and grade III becomes grade II.

Multivariate logistic regression analysis yielded two characteristics, out of the four investigated, which are needed to identify the definite MD ears versus the control ears, being cochlear PE and vestibular EH. Table 3 demonstrates our data broken down by these two criteria for the entire definite MD ears (78). Using solely the cochlear PE, a very high specificity was obtained of 97.4% (76/78), but the sensitivity was too low, i.e., 65.4% (51/78). This is because the grading “equal” yields a 50/50 MD versus control. So, this variable by itself is insufficient. However, by adding—as calculated by the logistic regression—the vestibular EH to an equal (in comparison with the normal ear) cochlear PE, the sensitivity increased significantly to 79.5% in case of the three-stage grading system. The specificity slightly decreased to 93.63% which is still very satisfactory. The introduction of the additional low-grade vestibular hydrops further increased the sensitivity from 79.5 to 84.6% without losing specificity (92.3%). The other variables (cochlear EH, vestibular PE) did not contribute significantly to this classification.

**Table 3:** Data of 78 patients with unilateral definite MD (78 definite MD ears and 78 normal control ears) broken down by the 2 characteristics yielded by logistic regression analysis, being cochlear PE and vestibular EH

Cochlear PE			MD or no MD		Total
			Control	Definite MD	
Less than contralateral	Vestibular EH 4-stage	Normal	47	2	49
		Grade 1	0	0	0
		Grade 2	3	1	4
		Grade 3	1	0	1
Equal	Vestibular EH 4-stage	Normal	21	9	30
		Grade 1	1	4	5
		Grade 2	1	5	6
		Grade 3	2	6	8
More than contralateral	Vestibular EH 4-stage	Normal	1	5	6
		Grade 1	0	2	2
		Grade 2	0	15	15
		Grade 3	1	29	30
	Total		78	78	156

*MD* Menière’s disease, *PE* perilymphatic enhancement, *EH* endolymphatic hydrops

For MD ears with exclusion of ITT (61 patients), the same tendency in sensitivity and specificity can be found. Cochlear PE remains the most specific parameter with a maximum increase in sensitivity by adding vestibular EH in a four-stage grading system (Table 4).

**Table 4:** Classification statistics for definite MD ears with inclusion and exclusion of ITT ears

MRI variables	Spec all definite MD (#78)	Sens all definite MD (#78)	Spec definite MD without ITT (#61)	Sens definite MD without ITT (#61)
Cochlear PE	97.4	65.4	97.4	62.3
Cochlear PE + Vestibular EH 3-stage	93.6	79.5	93.6	75.4
Cochlear PE + Vestibular EH 4-stage	92.3	84.6	92.3	82

*MD* Menière’s disease, *PE* perilymphatic enhancement, *EH* endolymphatic hydrops, *ITT* intratympanic therapy

In a following analysis, we correlated the duration of the MD illness with the different MRI-based variables. Cochlear and vestibular EH correlated significantly with the duration of the MD disease (Spearman rho = 0.23, *p* = 0.047 and rho = 0.32, *p* = 0.004, respectively). Also, cochlear PE correlated significantly with the duration of MD (rho = 0.28, *p* = 0.014).

## Discussion

Our study shows that delayed gadolinium-enhanced 3D-FLAIR is a reliable and accurate diagnostic technique with a high intra- and inter-reader agreement of, respectively, 0.88 and 0.79. These results are in line with previous studies with smaller patient populations [8]. In addition, this is the first study to report on the high intra-reader agreement of the technique.

We investigated the value of four variables in delayed gadolinium-enhanced 3D FLAIR MRI in patients with definite MD: that is cochlear EH, vestibular EH, cochlear PE, and vestibular PE. Multivariate logistic regression analysis yields only two discriminative parameters which are necessary to identify definite MD ears versus control ears: vestibular EH and cochlear PE.

### Modified four-stage vestibular EH grading system

Several different semi-quantitative MR grading systems for EH already exist.

This study shows that the commonly used vestibular semi-quantitative, three-stage EH grading system proposed by Baráth et al. [8] is reliable and that the addition of an extra low-grade vestibular EH, in which the saccule became equal or larger than the utricle but not yet confluent, increases sensitivity without loss of specificity. Accordingly, Attyé et al. described inversion of the saccule-to-utricle area ratio (SURI) as a qualitative marker [10].

In this study, the used 3D-FLAIR sequence did not show a consistent enhancement of the lateral semicircular canal, and thus we were not able to investigate the herniation of EH into the non-ampullated side of the lateral semicircular canal (Fig. 2d) nor in the common crus of the posterior semicircular canal. Recently, we added an isotropic 3D-FLAIR sequence to our protocol on which the semicircular canals invariably can be evaluated. It can be expected that with further increasing spatial resolution of the MRI sequence, the evaluation of the enlarged endolymphatic spaces into the semicircular canals can be done more reliably. The Nagoya group recently published their data on the occurrence of unilateral herniation into the semicircular canal with progression of EH, however with a very low incidence (3%) [11]. Herniation into the horizontal semicircular canal also has been reported to correlate with an impaired caloric response [12].

Cochlear EH, standard included in the Baráth grading system, does not seem to have an added predictive value in our study. This is in line with prior studies in which for the evaluation of patients with MD, only the evaluation of the saccule and utricle are incorporated. Moreover, the papers in literature on the use of heavily T2-weighted sequences in the evaluation of patients with definite MD also only evaluate the vestibule in demonstrating MD, showing a high percentage of dilated saccules in definite MD patients [13, 14].

### MRI of BPB impairment in MD

To the best of our knowledge, this is the first study to demonstrate the additional diagnostic value of the degree of cochlear PE in MD, being even highly specific. These results support the theory that there is an increased permeability of the blood-perilymph barrier (BPB) on the affected side of patients with unilateral MD. Increased PE in the affected inner ear of patients with MD has been noted in multiple imaging studies [6, 8, 15–17]. Tagaya et al. even noted the contrast effect correlated with the hydrops grade [17]. Also, transmission electron microscopic analysis of capillaries located in the normal human utricular stroma recently revealed differential ultrastructural pathological changes in Menière’s disease specimens, indicating that permeability alterations of the blood-labyrinth barrier may be one of the primary causes of endolymphatic hydrops in Menière’s disease [18].

Vestibular PE does not have an added value. This can be explained by the fact that in a grade 3 vestibular hydrops, by definition, there is no perilymphatic space left to evaluate.

As ITT might be a potential study bias, we analyzed both, the entire definite MD ears population and the subgroup of definite MD ears with exclusion of ITT. Our results demonstrate that ITT apparently does not change the classification statistics for cochlear PE, and probably does not influence the blood-perilymph-barrier.

It was hypothesized that the longer MD was present, the higher the grading of the EH staging would be, as well that the cochlear PE would be susceptible to the illness duration. As indicated in the results, all three variables were influenced by the duration of the disease.

### New imaging approach

**Fig. 4:**
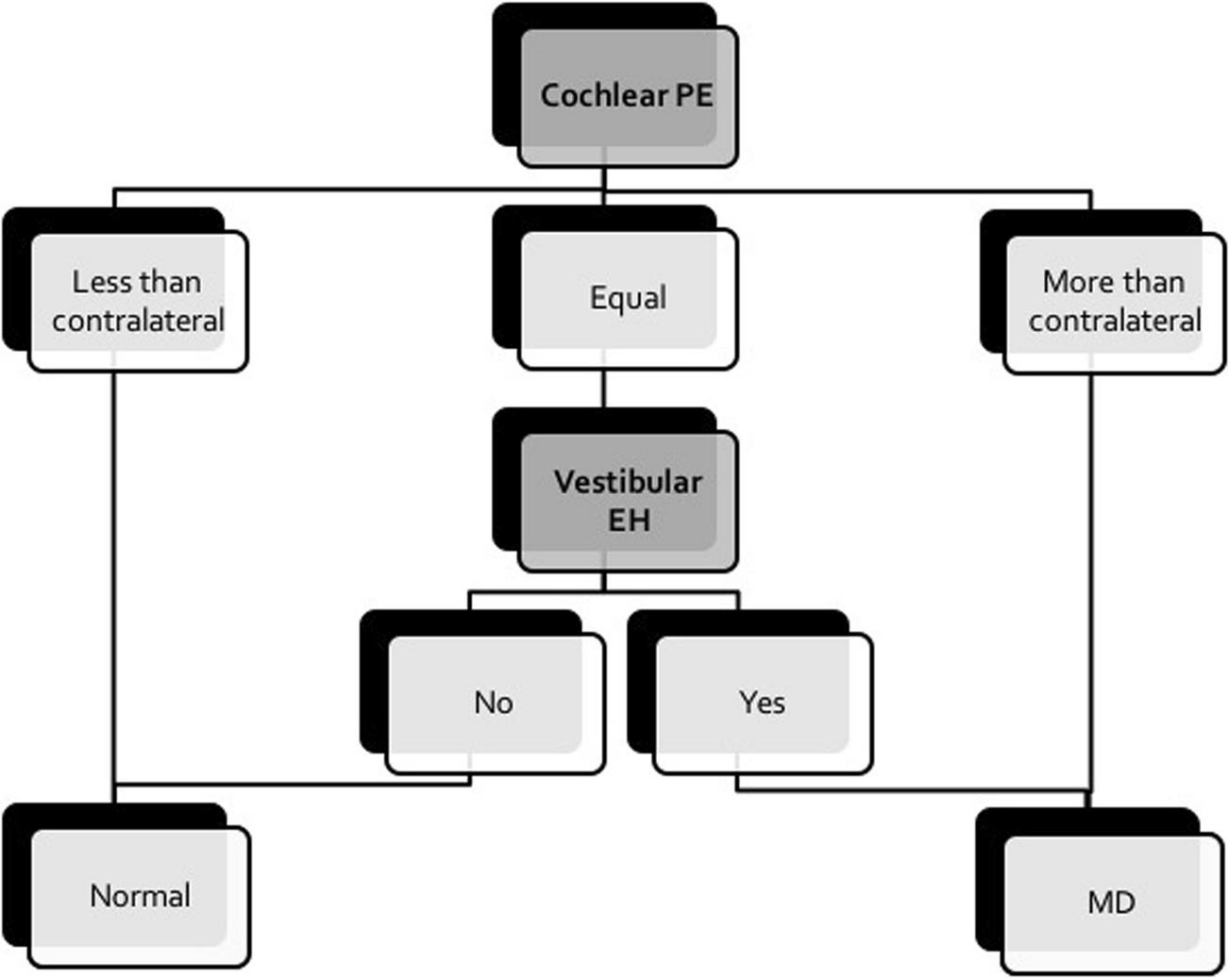
Proposed algorithm for workup of a unilaterally symptomatic ear which emerged from logistic regression analyses. If the cochlear PE is “less than contralateral,” the ear can likely be considered as normal. If cochlear PE is “equal,” the ear can be classified as normal when there is no vestibular hydrops. Otherwise when there is vestibular hydrops, the ear is highly suspicious of MD, even in case of a low-grade I vestibular hydrops. If cochlear PE is “more than contralateral,” the ear can be classified as MD, regardless of the absence or presence of vestibular EH

Our study findings suggest the following algorithm for workup of a unilaterally symptomatic ear (Fig. 4):

1. If the cochlear PE is “less than contralateral,” the ear can probably be considered as normal.
2. If the cochlear PE is “equal,” the evaluation of vestibular EH becomes decisive. In the absence of vestibular EH, the ear can be classified as normal. Otherwise, in the presence of vestibular EH, the ear can be classified as an MD ear, even in case of a low-grade I vestibular hydrops.
3. If the cochlear PE is “more than contralateral,” the ear can be classified as an MD ear, regardless of the presence of vestibular EH.

Obviously, these rules need to be confirmed in future prospective studies.

### Role of MRI in the future diagnostic criteria of MD

In the present study, the inter-reader agreement of the clinical evaluation was shown to be lower (0.66) than the MRI diagnostic criteria (0.79). This may indicate that MRI is a more consistent test than clinical assessment, and it may validate future incorporation of the MRI findings in the diagnostic criteria of MD to make them more robust. This is important because therapy of MD has far-reaching consequences, namely lifelong medication, or in severe cases, destruction of the membranous labyrinth.

### Limitations of this study

The major limitation of this study is that the contralateral clinically normal ear of the definite MD patients was used as a control group. Apart from the fact that in a retrospective study it is almost impossible to form such a matched control group, it is also for ethical reasons not allowed to inject a group of normal healthy volunteers with gadolinium in order to see the hydrops features in a normal population. Since the clinical, auditory, and vestibular functional tests for MD—based on the 2015 revised 1995 AAO-HNS criteria—are still considered the gold standard for the diagnosis of MD, and as in the majority of cases MD clinically can be confined to one ear, we could use the healthy contralateral normal ears as the control group. This was also the control group in the Baráth study.

In summary, the results of our study show that delayed gadolinium-enhanced 3D FLAIR MRI using vestibular-cochlear EH and PE grading system is a reliable technique. A four-stage vestibular EH grading system in combination with cochlear PE assessment gives the best diagnostic accuracy to detect MD. Cochlear EH and vestibular PE prove to have no additional diagnostic value.

## Abbreviations

MD: Menière’s disease
Gd: Gadolinium
MRI: Magnetic resonance imaging
EH: Endolymphatic hydrops
3D FLAIR: Three-dimensional fluid attenuated inversion recovery
PE: Perilymphatic enhancement
AAO HNS: American Academy of Otolaryngology, Head and Neck Surgery
ITT: Intratympanic therapy
BPB: Blood-perilymph barrier

## References

[1] AAO – HNS Committee on hearing and equilibrium guidelines for the diagnosis and evaluation of therapy in Menière’s disease. American Academy of Otolaryngology – Head and Neck Foundation, Inc (1995). Otolaryngol Head Neck Surg.

[2] Lopez-Escamez JA, Carey J, Chung WH, Goebel JA, Magnusson M, Mandalà M, Newman-Toker DE, Strupp M, Suzuki M, Trabalzini F, Bisdorff A (2015). Diagnostic criteria for Menière’s disease. J Vestib Res.

[3] Nakashima T, Pyykkö I, Arroll MA, Casselbrant ML, Foster CA, Manzoor NF, Megerian CA, Naganawa S, Young YH (2016). Meniere’s disease. Nat Rev Dis Primers.

[4] Hallpike CS, Cairns H (1938). Observations on the pathology of Ménière’s syndrome. Proc R Soc Med.

[5] Naganawa S, Nakashima T (2014). Visualization of endolymphatic hydrops with MR imaging in patients with Ménière’s disease and related pathologies: current status of its methods and clinical significance. Jpn J Radiol.

[6] Pakdaman MN, Ishiyama G, Ishiyama A, Peng KA, Kim HJ, Pope WB, Sepahdari AR (2016). Blood-labyrinth barrier permeability in Menière disease and idiopathic sudden sensorineural hearing loss: findings on delayed postcontrast 3D-FLAIR MRI. AJNR Am J Neuroradiol.

[7] Pyykkö I, Nakashima T, Yoshida T, Zou J, Naganawa S (2013). Meniere’s disease: a reappraisal supported by a variable latency of symptoms and the MRI visualization of endolymphatic hydrops. BMJ Open.

[8] Baráth K, Schuknecht B, Naldi AM, Schrepfer T, Bockisch CJ, Hegemann SC (2014). Detection and grading of endolymphatic hydrops in Menière disease using rtez. AJNR Am J Neuroradiol.

[9] Lane JI, Witte RJ, Bolster B, Bernstein MA, Johnson K, Morris J (2008). State of the art: 3T imaging of the membranous labyrinth. AJNR Am J Neuroradiol.

[10] Attyé A, Eliezer M, Boudiaf N, Tropres I, Chechin D, Schmerber S, Dumas G, Krainik A (2017). MRI of endolymphatic hydrops in patients with Meniere’s disease: a case-controlled study with a simplified classification based on saccular morphology. Eur Radiol.

[11] Sugimoto S, Yoshida T, Teranishi M, Kobayashi M, Shimono M, Naganawa S, Sone M (2018). Significance of endolymphatic hydrops herniation into the semicircular canals detected on MRI. Otol Neurotol.

[12] Gürkov R, Flatz W, Louza J, Strupp M, Ertl-Wagner B, Krause E (2012). Herniation of the membranous labyrinth into the horizontal semicircular canal is correlated with impaired caloric response in Ménière’s disease. Otol Neurotol.

[13] Venkatasamy A, Veillon F, Fleury A, Eliezer M, Abu Eid M, Romain B, Vuong H, Rohmer D, Charpiot A, Sick H, Riehm S (2017). Imaging of the saccule for the diagnosis of endolymphatic hydrops in Meniere disease, using a three-dimensional T2-weighted steady state free precession sequence: accurate, fast, and without contrast material intravenous injection. Eur Radiol Exp.

[14] Simon F, Guichard JP, Kania R, Franc J, Herman P, Hautefort C (2017). Saccular measurements in routine MRI can predict hydrops in Menière’s disease (2017). Eur Arch Otorhinolaryngol.

[15] Naganawa S, Kawai H, Taoka T, Suzuki K, Iwano S, Satake H, Sone M, Ikeda M (2016). Cochlear lymph fluid signal increase in patients with otosclerosis after intravenous administration of gadodiamide. Magn Reson Med Sci.

[16] Yamazaki M, Naganawa S, Tagaya M, Kawai H, Ikeda M, Sone M, Teranishi M, Suzuki H, Nakashima T (2012). Comparison of contrast effect on the cochlear perilymph after intratympanic and intravenous gadolinium injection. AJNR Am J Neuroradiol.

[17] Tagaya M, Yamazaki M, Teranishi M, Naganawa S, Yoshida T, Otake H, Nakata S, Sone M, Nakashima T (2011). Endolymphatic hydrops and blood-labyrinth barrier in Ménière’s disease. Acta Otolaryngol.

[18] Ishiyama G, Lopez IA, Ishiyama P, Vinters HV, Ishiyama A (2017). The blood labyrinthine barrier in the human normal and Meniere’s disease macula utricle. Sci Rep.

